# Circulating tumor cells are a good predictor of tumor recurrence in clinical patients with gastric cancer

**DOI:** 10.1038/s41598-024-63305-3

**Published:** 2024-06-04

**Authors:** Wenxing Li, Xin Zhang, Yanqi Yang, Jinhe Lin, Kai Zhou, Ruifang Sun, Chengxue Dang, Dongmei Diao

**Affiliations:** 1 Department of Surgical Oncology, Xi’an Jiaotong University Medical College First Affiliated Hospital, 277 West Yanta Road, Xi’an, 710061 Shaanxi China; 2 Department of Pathology, School of Basic Medical Sciences, Health Science Center, Xi’an Jiaotong University, Xi’an, 710061 Shaanxi China

**Keywords:** Gastric cancer, Circulating tumor cells, Metastasis, Overall survival, Disease-free survival, Cancer, Gastrointestinal cancer, Tumour biomarkers, Surgical oncology

## Abstract

Circulating tumor cells (CTCs) as a liquid biopsy have great potential in clinical applications and basic cancer research, but their clinical use in gastric cancer remains unclear. This study investigated whether CTCs could be used as a potential prognosis predictor in patients with gastric cancer. A total of 120 patients with pathologically confirmed gastric cancer were enrolled from January 1, 2015, to December 1, 2019. All patients were initially diagnosed without previous treatment, and then the number of CTCs was detected using the NEimFISH method before radical surgical resection. Regular follow-up was performed in all patients, and the correlations between the number of CTCs and clinical endpoints, such as disease-free survival (DFS) and overall survival (OS), were evaluated. The univariate and multivariate hazard ratios were calculated using the Cox proportional hazard model. Based on the number of CTCs, we defined CTCs ≥ 2 per 7.5 mL of whole blood as the positive group and CTCs < 2 as the negative group. Among the 120 patients who underwent CTC detection before surgery, the rate of CTC-positive patients was 64.17% (77/120) of which stage I and II patients accounted for 22.50% and stage III patients accounted for 41.67% (*P* = 0.014). By detecting CTCs before surgery and at the time of recurrence, the number of CTCs tends to increase concomitantly with disease progression (median: 2 VS 5 per 7.5 mL). Multivariate analysis showed that age (HR, 0.259; 95% CI, 0.101–0.662; *P* = 0.005), D-dimer (HR, 3.146; 95% CI, 1.169–8.461; *P* = 0.023), and lymph node metastasis (HR, 0.207; 95% CI, 0.0071–0.603; *P* = 0.004) were factors correlated with CTCs. In addition, the median follow-up of all the patients was 38.0 months (range of 28–80 months); the DFS in CTC-positive patients was significantly shorter than that of the CTC-negative patients, and a significant difference was found based on the Cox proportional hazard regression model analysis (44.52 ± 2.83 m vs. 74.99 ± 2.78 m, HR = 4.550, *P* = 0.018). The OS was shorter in the CTC-positive group than in the CTC-negative group before the operation, but the result was not significant based on the Cox proportional hazard regression model analysis (47.58 ± 2.46 m vs. 70.68 ± 3.53 m, HR = 2.261, *P* = 0.083). The number of CTCs tends to increase concomitantly with disease progression. In addition, the detection of CTCs was an independent predictor of shorter DFS in gastric cancer. However, the relationship between CTCs and OS needs to be determined in future studies.

## Introduction

Gastric cancer (GC) remains the fifth most common cancer and the third leading cause of cancer-related death worldwide^1^. According to the latest Japanese guidelines, surgery with postoperative adjuvant chemotherapy is still the standard treatment for patients with advanced GC^2^. Although some progress has been made in comprehensive therapy, immunotherapy, targeted therapy, and other treatments, given the aggressive biological invasiveness of tumors, late detection, and high disease progression or recurrence rate in patients with advanced tumors, their prognosis is still not optimistic, and their 5-year survival rate remains below 30%^3^. Approximately 50% of patients with GC have tumor recurrence or metastasis after radical surgical resection^4^. At present, ideal peripheral blood biomarkers, apart from tumor markers such as CEA and CA199, to guide treatment or disease monitoring are lacking. Therefore, identifying new, noninvasive, and better prognostic markers is urgently needed to facilitate diagnosis, predict prognosis, and determine treatment response in patients with GC^5^.

Metastasis is the main cause of high GC-related mortality rate^6^. Therefore, tumor cells likely circulate in the blood of most patients with GC at the time of diagnosis, regardless of any clinical evidence of distant metastasis. Although most shed tumor cells may die within the circulatory system because of physical and anatomical conditions, some circulating tumor cells (CTCs) exhibit a particularly malignant potential, acquire stem cell characteristics, and eventually evolve into metastases^7^. CTCs represent the phenotypic and genetic compositions of primary and metastatic tumors^8,9^. Recent studies have shown that CTCs are frequently found in many solid tumor types, such as lung^10^, breast^11^, esophageal^12^, bladder^13^, head and neck^14^, and colorectal cancers. Uenosono Y’s study found that CTC levels have a considerable connection with OS and DFS in patients with GC^15^. However, more evidence is needed to prove the prognostic role of CTC in gastric cancer.

For the diagnosis of CTCs, current methods rely on various techniques such as immunoaffinity-based methods that utilize specific protein markers on cancer cell surfaces, as well as differences in physical properties such as size, density, and electrical properties between cancer cells and blood cells. However, the CellSearch system, based on immunomagnetic cell enrichment, is a widely used techniques for the automated enrichment and detection of CTCs^16,17^. Epithelial-mesenchymal transition (EMT), an important process that occurs in CTCs, can reduces the expression level of epithelial surface markers^18^. However, systems relying on epithelial markers may fail to detect CTCs undergoing EMT^19^. In our study, the negative enrichment and immune fluorescence in situ hybridization (NEimFISH) technology was used to indirectly enrich CTCs by utilizing the expression of the common leukocyte antigen CD45 on peripheral blood leukocytes and removing irrelevant cells after binding with magnetic beads. This technology does not rely on the expression of antigens on the surface of tumor cells, making it applicable to a wide range of tumor types with a CTC recovery rate of over 90%. In addition, the iFISH technology was utilized, combining immunofluorescence detection with fluorescence in-situ hybridization techniques for CTC detection. As technology advances, next-generation microfluidic platforms with high sensitivity and specificity have been developed. CTCs were isolated using a helical channel microfluidic chip that separates cells of different sizes with different Dean forces in a curved channel. Consequently, CTCs that exhibit different expression levels of specific surface proteins can be captured, and the technique offers the advantages of fast isolation time, ease of use, and low cost^20^.

In this retrospective study, CTCs in primary diagnosed patients with GC were evaluated using the NEimFISH method, and all patients received radical GC surgery. Moreover, in this study, we aimed to evaluate CTCs in patients with primary gastric cancer before and after radical gastrectomy and on disease recurrence and investigated the predictive and prognostic role of CTCs in gastric cancer patients.

## Materials and methods

### Patients

This retrospective study was conducted at the Department of Oncology, First Affiliated Hospital of Xi’an Jiaotong University from January 2015 to December 2019. A total of 120 patients who underwent radical gastrectomy and had histologically proven adenocarcinoma of the stomach with a diagnosis of TNM stage I–III were included. The TNM stage was identified in accordance with the NCCN guidelines^21^. The exclusion criteria were previous treatment for GC and multiple primary cancers. At the same time, we collected blood samples of 10 healthy individuals from the health examination center, all of who had normal results in both laboratory and imaging examinations, and no signs of any diseases. Additionally, we also collected blood samples of 10 patients diagnosed with benign gastric tumors by histopathology, without chronic gastritis or other tumors. Based on the number of CTCs, we defined CTC ≥ 2 per 7.5 mL of whole blood as the positive group and CTC < 2 as the negative group using ROC analysis, which has been used in a previous study^22^. This study aimed to determine the frequency of CTC positivity in primary gastric cancer and to evaluate whether CTCs are a significant predictor of disease-free survival (DFS) in patients with gastric cancer. All patients were regularly followed up in our hospital every 3 months for the first 2 years post-operation and every 6 months for the following years. The patients also underwent endoscopy and computed tomography at least once a year, in accordance with their disease stage and course. All patients signed an ethically informed consent form (2015-046), and the study was approved by the Ethics Committee of the First Affiliated Hospital of Xi’an Jiaotong University. Informed consent was obtained from all subjects and/or their legal guardian(s). All methods were performed in accordance with the relevant guidelines and regulations.

### Methods

#### Specimen collection

7.5 mL of whole blood from the median cubital vein was taken before radical surgical resection. All blood samples were added to a citrate glucose anticoagulant vacuum blood collection tube, stored at room temperature (15–30 °C), and enriched within 24 h.

#### Enrichment by density gradient centrifugation

The kit contains reagents for removing peripheral blood leukocytes and fluorescence in situ hybridization (Cyttel Biosciences Inc., Jiangsu, China).

#### Separate plasma

7.5 mL of whole blood was taken into a 50 mL centrifuge tube, and CS1 (buffer solution) was added reaching 45 mL (whole blood: CS1 = 5:1). The sample was centrifuged at 650*g* for 5 min at room temperature (5920R, Eppendorf). Then, the supernatant was discarded to approximately 12 mL, and the precipitated cells were resuspended by gently shaking the centrifuge tube.

#### Lysis of red blood cells

CS2 (red blood cell lysis solution) was added reaching 45 mL and centrifuged at 650*g* for 5 min at room temperature. The supernatant was discarded, leaving approximately 100–500 μL, and the precipitated cells were resuspended by gently shaking the centrifuge tube.

#### Washing of magnetic beads

An appropriate amount of magnetic bead suspension (150 μL per sample) was drawn into a 2 mL EP tube and place on a magnetic rack for about 1 min. After the solution becomes clear, the clarified solution was discarded. Then, 1 mL of CS1 was added to resuspend the beads, and the solution stayed on the magnetic rack for 1 min. Afterward, the clarified solution was discarded. The washing process was repeated three times, and then the beads were resuspended using 150 μL of CS1.

#### Removal of white blood cells

150 μL of magnetic beads was gradually added into the sample, and the centrifuge tube was fixed at a 40° angle on a horizontal shaker and shaken at room temperature at 100 rpm for 20 min. 3 mL of CS3 (separation medium) was added to a 50 mL centrifuge tube, and all the liquids from the magnetic beads were gently poured onto the top of the CS3 layer. The solution was centrifuged at 650*g* for 5 min at room temperature. The top layers of the solution were carefully aspirated. CS1 was added to the solution reaching 14 mL and centrifuged (950*g* for 5 min at room temperature). Then, the supernatant was discarded to 300 μL, and 1 mL of CS1 was added. The sample was placed on a magnetic rack for 2–3 min and centrifuged (2070*g* for 3 min at room temperature). Finally, the supernatant was discarded leaving 100 μL, and 100 μL of CCF1 (cell fixative solution) was added.

#### Smear and dry

The specimen was smeared on a 20 mm × 20 mm specimen frame and air dried. FISH processing was performed within 24 h after smearing.

#### Immunofluorescence staining

A total of 200–300 μL of CF2 (cell fixative solution) fixative was used to completely cover the specimen area, and fixed at room temperature for 8 min. CF2 was removed from the specimen area and placed in a preheated staining jar I (Saline-Sodium Citrate Buffer, 2× SSC) for 10 min. Each specimen slide was allowed to stand in the staining jar III (75% ethanol), IV (85% ethanol), and V (anhydrous ethanol) for 2 min and air-dried at room temperature. Afterward, 10 μL of the mixture of Centromere probe 8 (CEP8) and Centromere probe 17 (CEP17) was added to each specimen area, and the specimen was covered with a coverslip. Then, the edges of the coverslip were completely sealed with 200–300 μL of Rubber Cement for hybridization: denaturation was performed at 76 °C for 5 min, and hybridization was conducted at 37 °C for 1.5 h before washing the slides (preheated formamide solution, pH 6.7–7.2). The antibody working solution was prepared by mixing 20 μL of CD45-AF594 fluorescent antibody (Gene Tex, mouse, monoclonal, diluted 1:100) and 180 μL of 2% BSA (Pioneer, Xi’an, China) solution per sample. The specimen area was washed two times with 0.2% BSA (300 μL each time). Then, 0.2% BSA was removed from the specimen area, and the prepared antibody was added and incubated at room temperature for 1 h in the dark. Subsequently, 10 μL of DAPI (5 mg/mL, BD5010, Bioworld Technology, Co, Ltd., Nanjing, China) was added to the specimen area. The specimen was covered with a coverslip. The cells were observed under a fluorescence microscope (BX53, Olympus, Tokyo, Japan) and stored in the dark at 2–8 °C for 1 week.

#### Typical CTC criteria

**Figure 1:**
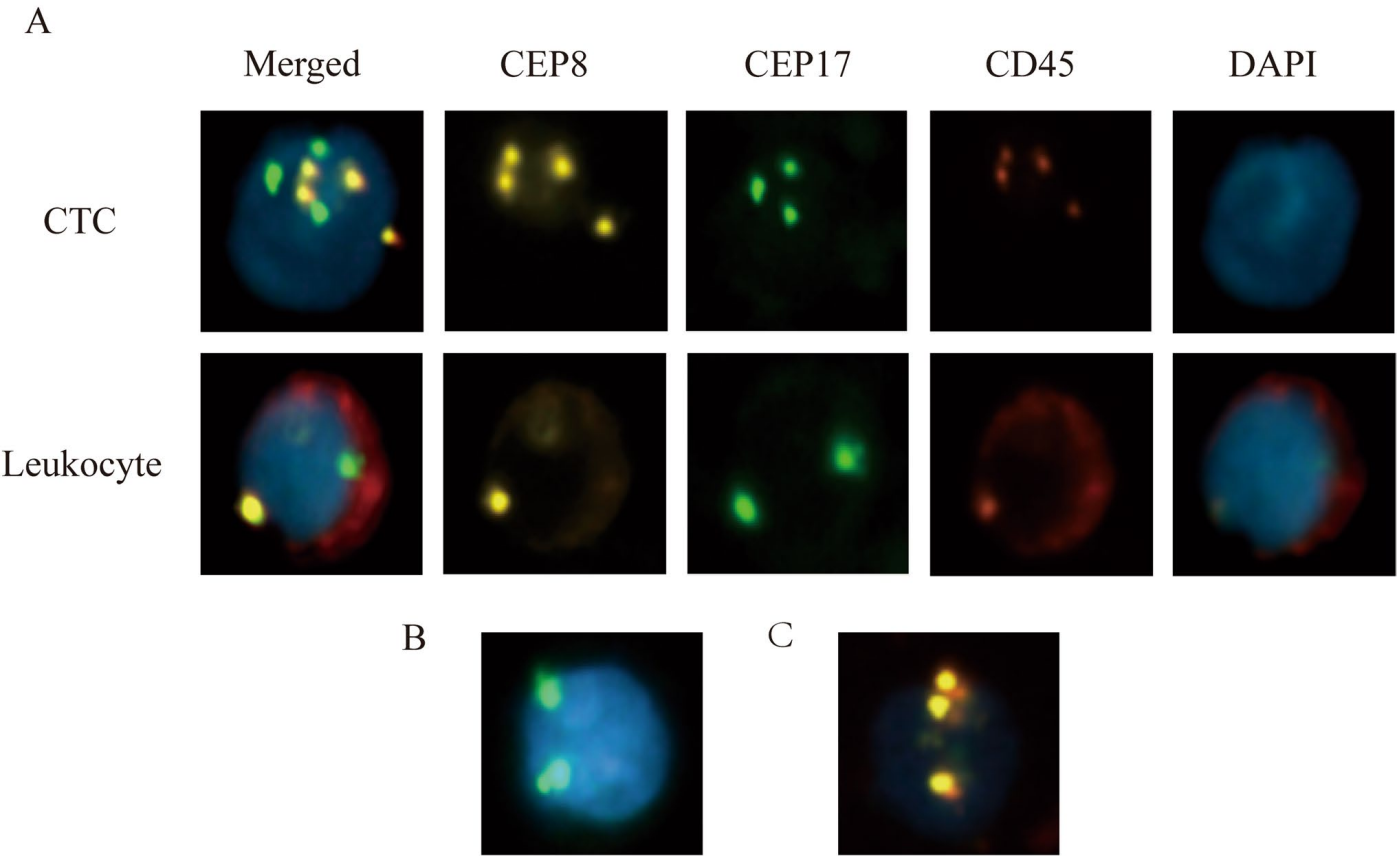
Images of CTCs and leukocytes detected by the NEimFISH method. (**A**) 40× CEP8 tetraploid/CEP17 triploid, CD45 (−), DAPI (+); (**B**) 40× CEP17 triploid, CD45 (−), DAPI (+); (**C**) 40× CEP8 triploid, CD45 (−), DAPI (+).

(1) The individual cells have homogeneous cytoplasm, and no layering is observed. (2) Three or more signals are present, and they are located in the cell nucleus. (3) CD45 staining is not observed in the red channel. (4) There are no magnetic beads are found on the cell surface. After identification, the cells that meet the above criteria can be epithelial tissue-derived tumor cells, namely, CTCs (Fig. 1).

### Statistical analysis

Categorical data were compared using the χ^2^ test, and continuous variables were compared using Student’s *t* test. Two-sided *P* values < 0.05 were considered statistically significant. Multivariate analysis was performed to compare the CTC-negative and CTC-positive groups using logistic regression models to estimate the odds ratio for each factor. For survival analyses, Cox proportional hazard models were used for univariate and multivariate analyses, and the hazard ratio (HR) was estimated. All statistical analyses were performed using SPSS Statistic 25.0.

### Ethics approval and consent to participate

This study was approved by the Ethics Committee of First Affiliated Hospital of Xi’an Jiaotong University. The patients provided their written informed consent to participate in this study.

## Results

### Patient characteristics

**Figure 2:**
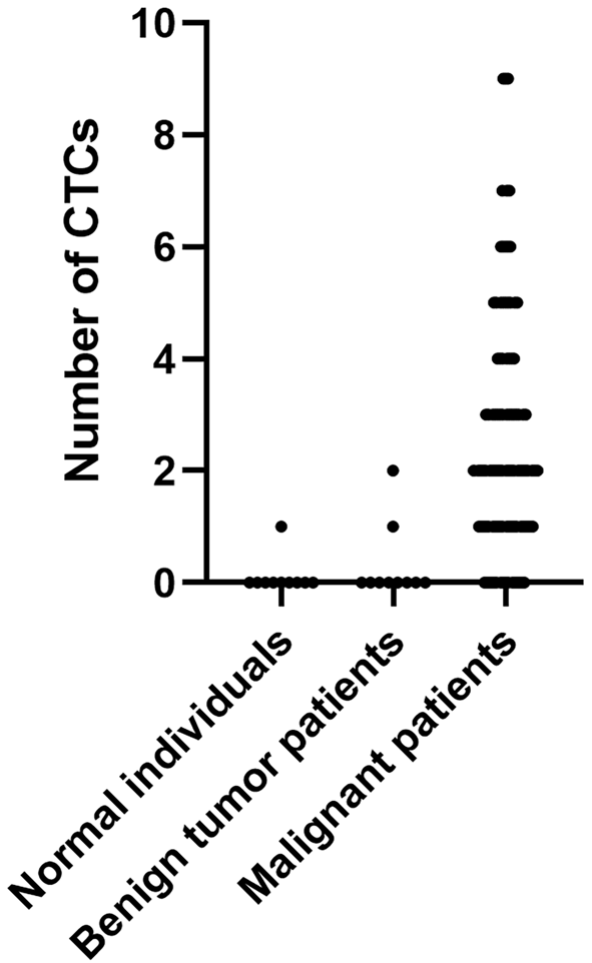
CTCs detection results in normal individuals, benign patients, and gastric cancer patients.

**Figure 3:**
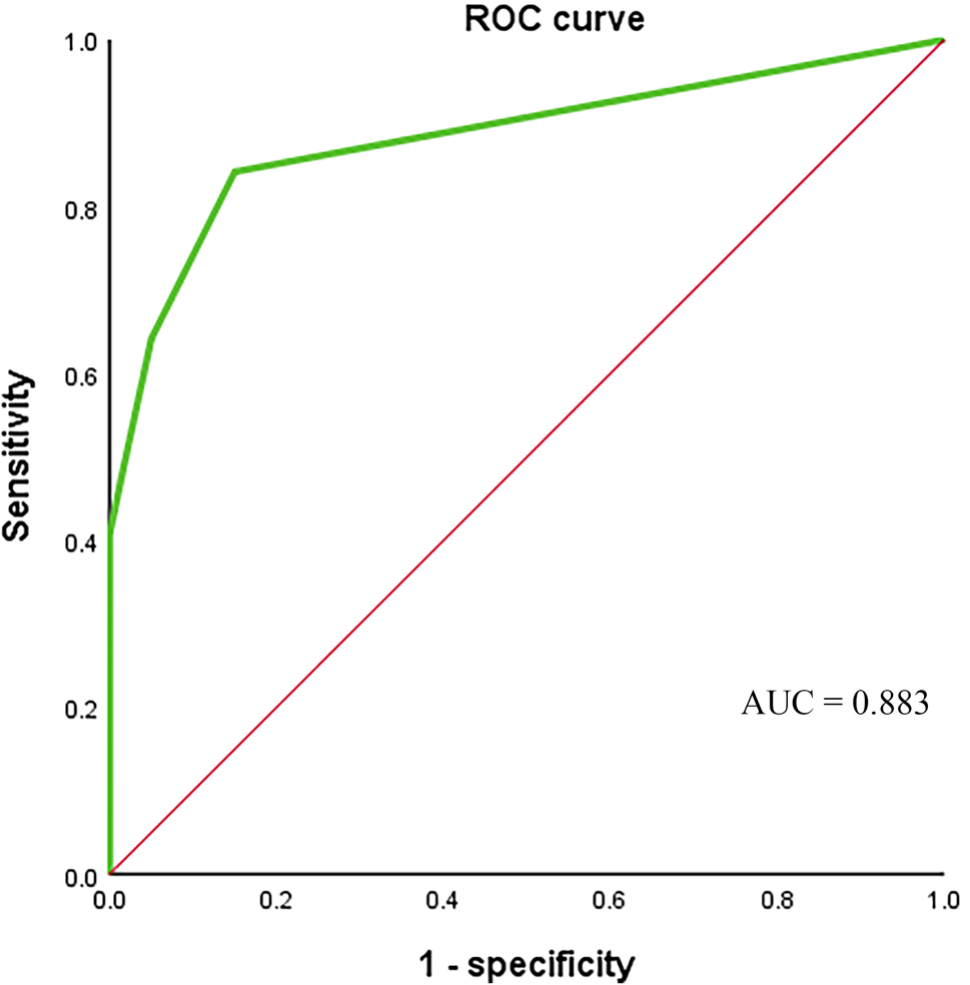
ROC analysis of CTCs detection in normal individuals, benign, and malignant patients.

**Figure 4:**
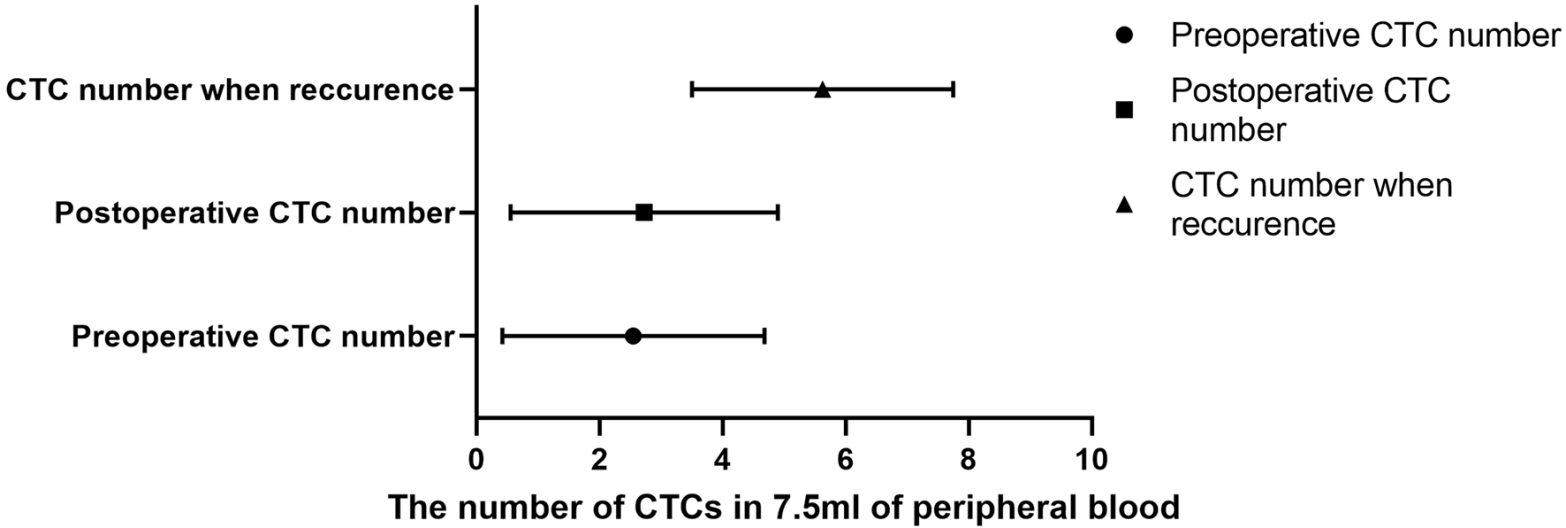
The mean CTC count at each time-point (preoperative, postoperative, on disease recurrence).

**Figure 5:**
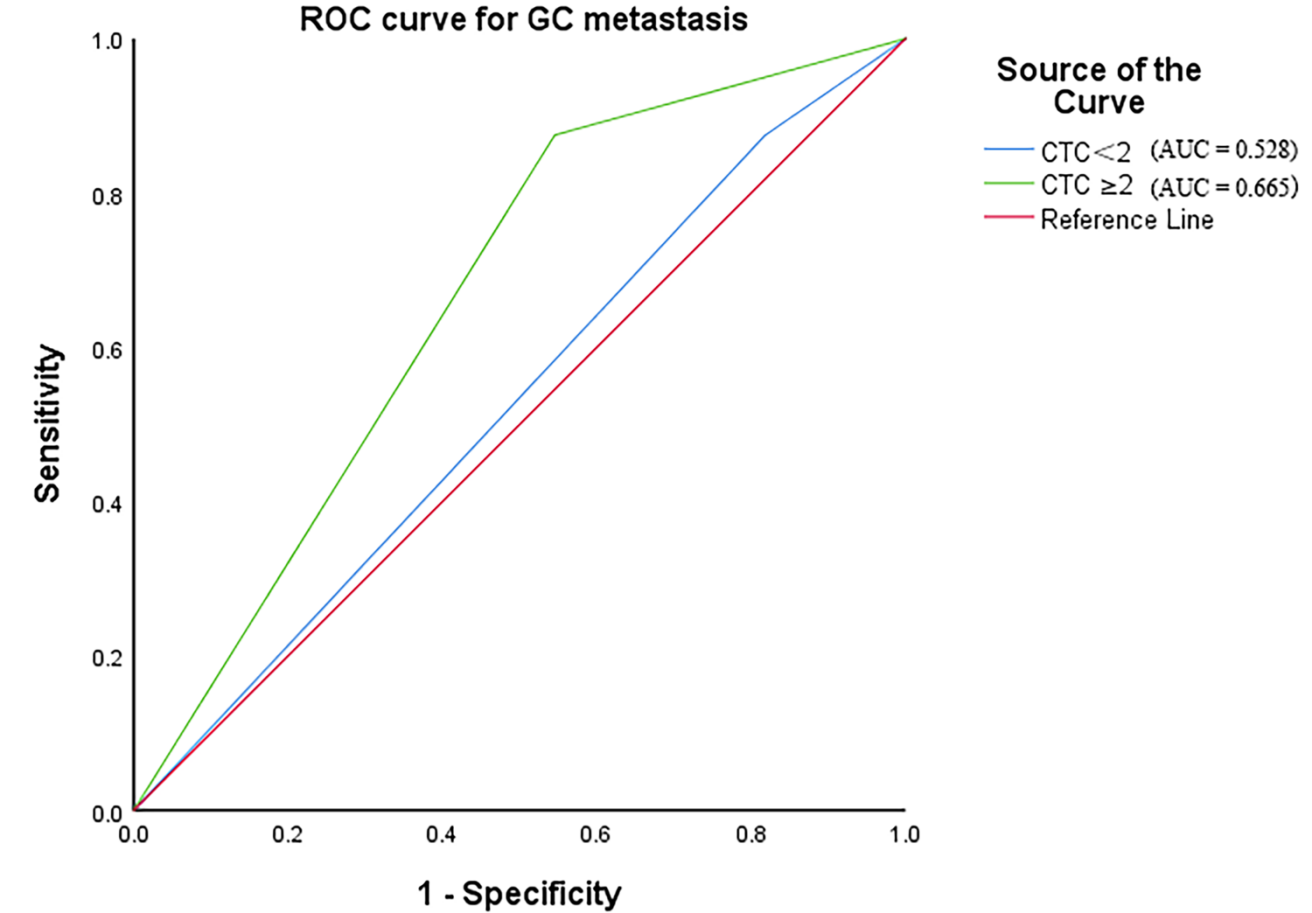
ROC analysis of GC metastasis. AUC indicates the diagnostic power of CTC < 2, CTC ≥ 2 and prediction probability for GC metastasis.

A total of 120 patients with histologically or cytologically confirmed GC with TNM stage I–III gastric cancer (26 males and 94 females; mean age, 60 years; age range, 32–80 years) who met the inclusion criteria between January 1, 2015, and December 1, 2019, and underwent radical gastrectomy in the Department of Oncology, First Affiliated Hospital of Xi’an Jiaotong University were used as the subjects of this study. In addition, 10 cases of benign gastric tumors and 10 healthy volunteers were collected. In this study, CTC counts were performed using the NEimFISH method. The positive detection rates in healthy individuals, benign gastric tumors, and GC were 0%, 10% (1/10), and 64.17% (77/120), respectively (Fig. 2). Based on ROC analysis, the area under the curve (AUC) was 0.883, indicating the good specificity of this detection method (Fig. 3). The relationship between the status of CTCs and the clinical factors is summarized in Table 1, and the number of CTCs detected in each patient is shown in Supplement Table 1. The preoperative CTCs of 120 patients with GC were examined, with the mean CTC count of 2.55 per 7.5 mL. Moreover, CTC reassessment was conducted in 40 of these patients 3 months after surgery, revealing the mean CTC count of 2.73 per 7.5 mL. Furthermore, in the group of patients who experienced recurrence, the CTC count at the time of recurrence indicated a mean of 5.63 per 7.5 mL (Fig. 4). Based on the number of CTCs, we defined CTCs ≥ 2 as the positive group and CTCs < 2 as the negative group. ROC analysis was performed to evaluate the diagnostic efficacy of CTC counts for predicting GC metastasis. The AUC of CTCs ≥ 2 for predicting GC metastasis was 0.665 (95% confidence interval [CI]: 0.553–0.777, *P* = 0.014) and that of CTCs < 2 was 0.528 (95% CI: 0.401–0.656, *P* = 0.671, Fig. 5). Among the 120 patients who underwent CTC detection before surgery, the positive rate of CTCs was 64.17% (77/120), of which stage I and II patients accounted for 22.50% and stage III patients accounted for 41.67% (*P* = 0.014). A positive CTC count was more common in tumors with a high T stage (*P* = 0.026), the presence of lymph node metastasis (*P* < 0.001), and a high level of serum CEA (*P* = 0.023). Cancer embolus (*P* = 0.023) and neural invasion (*P* = 0.003) were also common in CTC-positive patients.

**Table 1:** The relationships between the CTC status and clinical factors (n = 120).

Clinical factors	CTCs		P
	Negative (n = 43)	Positive (n = 77)	
Gender			0.125
Man	6	20	
Woman	37	57	
Age (year)			0.047^a^
≤ 60	17	45	
> 60	26	32	
BMI (kg·m^−2^)			0.169
< 24	23	51	
≥ 24	20	26	
D-dimer (ng/mL)			0.043^a^
< 1	31	41	
≥ 1	12	36	
CEA (ng/mL)			0.023^a^
< 3.4	33	43	
≥ 3.4	10	34	
CA199 (ng/mL)			0.301
≤ 39	34	62	
> 39	3	11	
CA724 (ng/mL)			0.396
≤ 6.9	29	54	
> 6.9	5	15	
Tumor location			0.742
Cardia	17	34	
Body	14	20	
Pylorus	12	23	
Histology grade			0.870
G1	3	5	
G2	17	27	
G3	23	45	
TNM stage			0.014^a^
I + II	25	27	
III	18	50	
T stage			0.026^a^
T1 + T2	21	22	
T3 + T4	22	55	
Lymph node metastasis			< 0.001^b^
Negative	33	30	
Positive	10	47	
Embolus			0.023^a^
Negative	33	43	
Positive	10	34	
Neural invasion			0.003^b^
Negative	34	49	
Positive	9	37	

*BMI* body mass index, *TNM* tumor-node-metastasis. All serological indicators were collected before surgery.

^a^P < 0.05 and ^b^P < 0.01.

### Multivariate analysis of the relationships between the CTC status and clinical factors

The relationship between the status of CTCs and clinical factors was also analyzed. Multivariate analysis using the log-rank test showed that age (HR, 0.259; 95% CI, 0.101–0.662; P = 0.005), D-dimer (HR, 3.146; 95% CI, 1.169–8.461; P = 0.023), and lymph node metastasis (HR, 0.207; 95% CI, 0.0071–0.603; P = 0.004) were the factors correlated with CTCs. The results are shown in Table 2.

**Table 2:** Multivariate analysis of the relationships between the CTC status and clinical factors.

Clinical factors	*B*	SE	Wald	Sig	HR	95% CI
Age (≤ 60 vs. > 60)	− 1.352	0.480	7.943	0.005^b^	0.259	0.101–0.662
D-dimer (< 1 vs. ≥ 1)	1.146	0.505	5.153	0.023^a^	3.146	1.169–8.461
CEA (< 3.4 vs. ≥ 3.4)	0.251	0.516	0.236	0.627	1.285	0.467–3.533
T stage (T1 + T2 vs. T3 + T4)	− 0.482	0.581	0.687	0.407	0.617	0.198–1.930
Lymph node metastasis (N vs. P)	1.746	0.562	9.662	0.002^b^	5.731	1.906–17.232
Embolus (N vs. P)	− 0.029	0.593	0.002	0.961	0.971	0.304–3.103
Neural invasion (N vs. P)	1.002	0.616	2.645	0.104	2.724	0.814–9.111

*B* beta, *SE* std. error, *df* degree of freedom, *Sig.* significance test, *HR* hazard ratio, *CI* confidence interval. The references for D-dimer, CEA, T stage, lymph node metastasis, embolus, and neural invasion were as follows: D-dimer < 1 ng/mL, CEA < 3.4 ng/mL, T stage I + II, lymph node metastasis negative, embolus negative, and neural invasion negative. *N* negative, *P* positive. ^a^P < 0.05 and ^b^P < 0.01.

### Risk factors for disease-free survival after curative surgery

We followed up 120 patients after surgery. All but eight patients were followed successfully until their death or recurrence. The follow-up rate was 93.33%. Patients who were followed up for less than 6 months were not included in the survival analysis. At the time of analysis, 24 of the 112 (21.43%) evaluable patients had experienced disease progression. Among them, 21 patients with positive CTCs had recurrence, and three patients with negative CTCs had recurrence. The median CTC counts before treatment and at disease progression were 2 and 5 per 7.5 mL, respectively. Factors increasing the risk of relapse were also investigated by Cox proportional hazard analysis. Univariate analysis showed that preoperative CTC positivity (P = 0.007), D-dimer ≥ 1 ng/mL (P = 0.007), TNM stage III (P = 0.001), and embolism (P = 0.017) were significant risk factors for DFS. However, multivariate Cox analysis indicated that preoperative CTC positivity (44.52 ± 2.83 m vs. 74.99 ± 2.78 m, HR = 4.550, P = 0.018) and TNM stage III (HR, 4.512; 95% CI, 1.141–17.846; P = 0.032) were significant risk factors for DFS. The results are shown in Table 3.

**Table 3:** Univariate and multivariate analysis of clinical factors for disease-free survival (N = 112).

Clinical factors	Univariate analysis			Multivariate analysis		
	HR	95% CI	P	HR	95% CI	P
Age (year)			0.051			0.109
≤ 60	1 (Ref)			1 (Ref)		
> 60	2.327	0.995–5.440		2.079	0.848–5.094	
Gender			0.956			
Woman	1 (Ref)					
Man	0.973	0.363–2.607				
Preoperative CTCs			0.007^b^			0.018^a^
Negative	1 (Ref)			1 (Ref)		
Positive	5.399	1.598–18.246		4.550	1.293–16.010	
Postoperative CTCs			0.261			
Negative	1 (Ref)					
Positive	2.432	0.516–11.465				
D-dimer (ng/mL)			0.007^b^			0.114
< 1	1 (Ref)			1 (Ref)		
≥ 1	3.253	1.385–7.639		2.186	0.829–5.764	
CEA (ng/mL)			0.092			
< 3.4	1 (Ref)					
≥ 3.4	1.993	0.849–4.444				
CA199 (ng/mL)			0.657			
≤ 39	1 (Ref)					
> 39	1.320	0.387–4.507				
TNM stage			0.001^b^			
I + II	1 (Ref)					
III	7.123	2.121–23.929				
Embolus			0.017^a^			0.852
Negative	1 (Ref)			1 (Ref)		
Positive	2.689	1.193–6.063		1.093	0.431–2.772	
Neural invasion			0.053			0.716
Negative	1 (Ref)			1 (Ref)		
Positive	2.212	0.990–4.941		0.833	0.312–2.227	
Chemotherapy			0.096			
Yes	1 (Ref)					
No	2.490	0.850–7.292				

*HR* hazard ratio, *CI* confidence interval, *Chemotherapy* postoperative adjuvant chemotherapy. ^a^P < 0.05 and ^b^P < 0.01.

### Risk factors for overall survival after curative surgery

At the time of analysis, 25 of the 112 (22.32%) evaluable patients had succumbed to their diseases. The number of deaths in patients with positive CTCs before surgery was 19, and that in patients with negative CTCs was 6. Univariate analysis showed that age > 60 years (P = 0.038), TNM stage III (P = 0.001), D-dimer ≥ 1 ng/mL (P = 0.001), existence of embolism (P = 0.008), neural invasion (P = 0.034), and chemotherapy (P = 0.033) were significant risk factors for OS. However, multivariate Cox proportional hazard analysis indicated that D-dimer ≥ 1 ng/mL (HR, 2.770; 95% CI, 1.097–6.994; P = 0.031) and TNM stage III (HR, 7.066; 95% CI, 1.367–36.535; P = 0.020) were significant risk factors for overall survival (OS). The results are shown in Table 4.

**Table 4:** Univariate and multivariate analysis of clinical factors for overall survival (N = 112).

Clinical factors	Univariate analysis			Multivariate analysis		
	HR	95% CI	P	HR	95% CI	P
Age (year)			0.038^a^			0.293
≤ 60	1 (Ref)			1 (Ref)		
> 60	2.434	1.050–5.642		1.590	0.670–3.774	
Gender			0.285			
Woman	1 (Ref)					
Man	0.621	0.259–1.488				
Preoperative CTCs			0.083			
Negative	1 (Ref)					
Positive	2.261	0.898–5.689				
Postoperative CTCs			0.316			
Negative	1 (Ref)					
Positive	2.998	0.350–25.666				
D-dimer (ng/mL)			0.001^b^			0.031^a^
< 1	1 (Ref)			1 (Ref)		
≥ 1	4.249	1.765–10.229		2.770	1.097–6.994	
CEA (ng/mL)			0.338			
< 3.4	1 (Ref)					
≥ 3.4	1.471	0.668–3.241				
CA199 (ng/mL)			0.669			
≤ 39	1 (Ref)					
> 39	1.307	0.383–4.463				
TNM stage			0.001^b^			0.020^a^
I + II	1 (Ref)			1 (Ref)		
III	11.431	2.693–48.524		7.066	1.367–36.535	
Embolus			0.008^b^			0.825
Negative	1 (Ref)			1 (Ref)		
Positive	2.977	1.335–6.639		1.105	0.456–2.678	
Neural invasion			0.034^a^			0.967
Negative	1 (Ref)			1 (Ref)		
Positive	2.348	1.065–5.174		0.981	0.401–2.397	
Chemotherapy			0.033^a^			0.649
Yes	1 (Ref)			1 (Ref)		
No	3.708	1.109–12.399		1.348	0.372–4.885	

*HR* hazard ratio, *CI* confidence interval, *Chemotherapy* postoperative adjuvant chemotherapy. ^a^P < 0.05 and ^b^P < 0.01.

### Circulating tumor cells are a good predictor of tumor recurrence in clinical patients with GC

**Figure 6:**
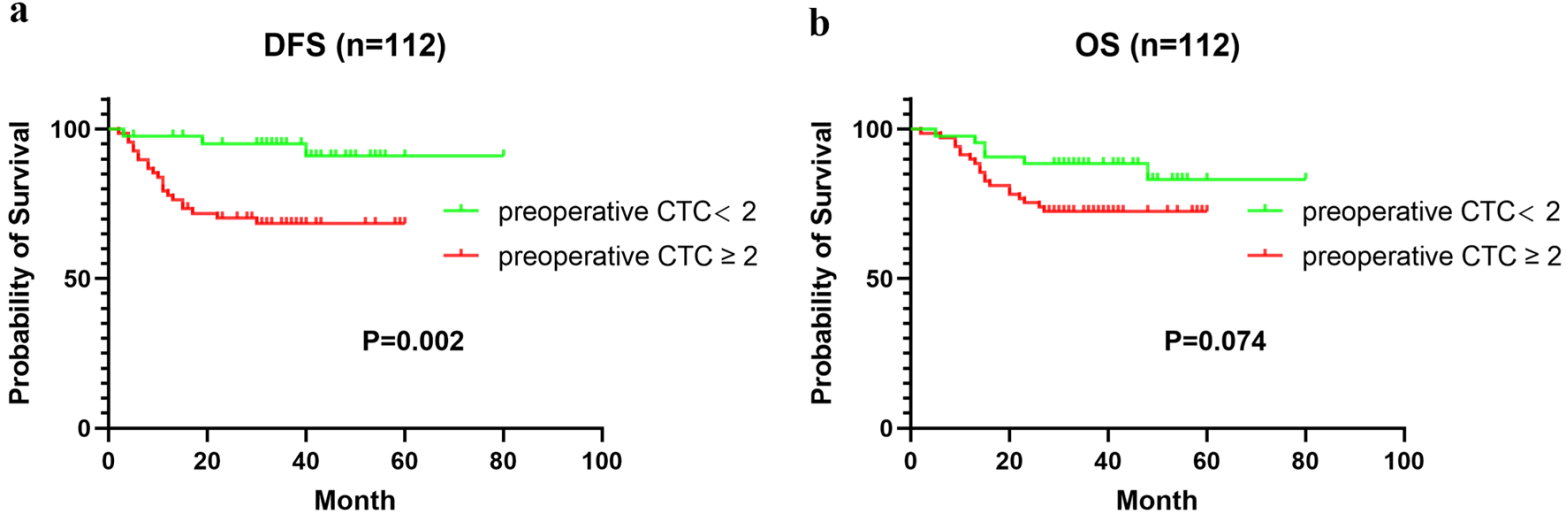
Kaplan–Meier survival curves of CTC < 2 and CTC ≥ 2 groups for DFS (**a**) and OS (**b**).

Through Kaplan–Meier analysis, we found that the DFS time of CTC-positive patients before surgery was significantly shorter than that of CTC-negative patients (44.52 ± 2.83 m vs. 74.99 ± 2.78 m, χ^2^ = 9.253, *P* = 0.002). However, OS was shorter in the CTC-positive group than in the CTC-negative group before the operation, but the difference was not significant (47.58 ± 2.46 m vs. 70.68 ± 3.53 m, χ^2^ = 3.195, *P* = 0.074). The results are shown in Fig. 6. Because the disease-free survival rate and overall survival rate among stage I, II and III are various, we also conducted subgroup analysis. The Kaplan–Meier analysis showed that there was no statistical differences in DFS and OS among stage I,II gastric patients between CTC ≥ 2 and CTC < 2 (*P* > 0.05) and the same results can also be observed in the OS of stage III gastric patients. However, we found that patients with CTC ≥ 2 showed a poorer DFS in stage III (P = 0.013), as shown in Supplement Fig. 1. Moreover, repeat analyses using a CTC cut-off of 5/7.5 mL showed that patients with a preoperative CTC count ≥ 5 had worse DFS and OS (Supplement Fig. 2).

## Discussion

In this retrospective study, we defined CTCs ≥ 2 per 7.5 mL of whole blood as the positive group and CTCs < 2 as the negative group using ROC analysis as shown in Fig. 5. CTCs were detected in 64.17% (77/120) of patients with GC of which stage I and II patients accounted for 22.50% and stage III patients accounted for 41.67%. By detecting CTCs before surgery and at the time of recurrence, we found that the number of CTCs tends to increase concomitantly with disease progression (median: 2.55 vs. 5.63 per 4.5 mL). Our detection method has demonstrated good specificity. Yang et al.^22^ applied the imFISH analysis in the study of lung cancer, gastric cancer, breast cancer, and esophageal cancer. They found that when using CTC ≥ 2 as the cut-off value, the positive detection rates of tumors were 71.33%, 86.21%, 76.77%, and 78.35%, indicating high sensitivity and relatively low false positives. This study showed a statistically significant difference (*P* < 0.05) between the preoperative CTC detection count and the survival rate of patients with gastric cancer. Using a detection cut-off value of ≥ 2, the survival rates significantly decreased. This result is consistent with previous research findings^23,24^.

Our previous preliminary study showed that the number of CTCs isolated from patients with cancer was related to surgical and pathological disease progression. In general, patients with positive CTCs were older than those with negative CTCs. The above-mentioned result may be because elderly patients are more likely have micro-metastases because of their low immunity. As the growth of the age, the immune system gradually declines, and is characterized by the number of immune cells and functional decline^25^. This can lead to reduced immune surveillance and clearance capabilities, facilitating the entry of tumor cells into the circulation and rendering them less susceptible to immune elimination. In addition, the elderly are predisposed to chronic disease or a state of chronic inflammation that may perpetuate immune activation while compromising immune functionality^26^. Such an environment may favour tumor proliferation and dissemination, thereby increasing the detection rate of CTCs in the bloodstream. Similarly, CTC-positive patients showed higher CEA levels. CEA is an acidic glycoprotein that is widely present on the surface of tumor cells, and its expression level can reflect the number of tumor cells in the body to a certain extent^27^. The content of serum CEA increases significantly with the occurrence and development of gastric cancer; simultaneously, the proliferation, differentiation, and metastasis of tumor cells will lead to an increase in the amount of tumor shedding^28^. Therefore, a significant correlation was found between CTC and CEA.

Based on the relationship between CTCs and a high coagulation index, CTCs and the hypercoagulability index D-D were significantly and positively correlated; that is, the D-D level of CTC-positive patients was significantly higher than that of CTC-negative patients. Some studies have also confirmed this result. In Yanhua Jiao’s research on the relationship between lung cancer CTCs and blood coagulation, he found that the levels of D-D, FIB, PT, and PLT were significantly higher in CTC-positive patients than in CTC-negative patients (all P < 0.05)^29^. Under certain conditions, tumor cells can secrete and release tissue factors, pro-coagulant factors, etc., thereby activating prothrombin and coagulation processes so that the body is in a hypercoagulable state^7,30^. In addition, the hypercoagulable state of blood promotes the ability of tumor emboli to adhere to the vessel wall of target organs, thereby promoting tumor metastasis^31,32^.

According to relevant research reports, the number of CTCs in peripheral blood is closely related to the TNM staging and T and N stages in GC^33^. A high N stage indicates the presence of cancer cells in the lymph nodes or the lymphatic vessels surrounding the tumor, and these cancer cells likely enter the peripheral blood system through the lymphatic circulation; thus, the higher the N stage is, the higher the number of detected CTCs. With the continuous increase in T stage, the tumor gradually infiltrates all layers of the esophagus, and tumor cells can enter the circulatory system through the lymphatic and blood reflux of all layers of the gastric wall^34^.

In addition, we found that the number of CTCs was related to clinical disease stage and relapse after curative surgery in patients with GC. In the current stage of research, compared with patients with one or fewer CTCs, the incidence of relapse-free survival in patients with more than one CTC is significantly lower, which is consistent with our findings^23^. The OS rate of patients with more than one CTC tended to be lower than that of patients with one or less; however, the difference was not statistically significant. Our research showed that the number of CTCs was not an independent risk factor of OS. In contrast to the results of some studies, the abovementioned results may be due to the relatively small sample size of our study, which is a single-center study and needs further study to verify the impact of CTCs on OS.

The CTC levels of 40 patients were also detected 1 month after surgery, and the postoperative CTC levels were generally higher than those before surgery, but survival analysis showed that postoperative CTC levels could not predict the prognosis of patients (Supplement Fig. 3). In addition, according to the number of postoperative CTCs more than, less than and equal to the number of preoperative CTCs, the patients are categorized into elevated, reduced and unchanged groups, respectively. Comparing their effects on the postoperative prognosis of GC, we found that dynamic changes in postoperative CTCs did not predict patient survival (Supplemental Fig. 4), which may be due to the small sample size. Most of the enrolled patients had middle-advanced GC, and selection bias was observed. Therefore, a large number of samples are necessary to verify the effect of postoperative CTC levels on prognosis.

At present, the most common approach for capturing CTCs is the cell search system, which is based on the expression of the adhesion molecule EpCAM on the tumor cell surface and cytokeratins (CKs)^35–37^. However, isolation and identification are challenging because of the rarity of CTCs in the blood circulation and their molecular and phenotypic heterogeneity^37^. However, the expression of EPCAM has high heterogeneity and different dynamics among different types of epithelial tumor cells, and the transliteration of epithelial intermediate transformation (EMT) may reduce the expression level of EPCAM and CKS^18^. Therefore, this method may not accurately detect CTCs. A new technology, namely, NEimFISH, combines negative enrichment and immune fluorescence in-situ hybridization (imFISH), and it can improve the sensitivity and specificity of CTC detection by detecting genes and proteins simultaneously^38^. The NEimFISH method can improve the sensitivity and specificity of CTC detection^39^. Previous studies have shown that iFISH has a higher detection rate of CTCs in patients with GC^40^ or pancreatic cancer^41^. This innovative technology ensures the accuracy of our experimental data and enhances the reliability of the results^39^. Thus, in this study, the NEimFISH method was used to detect CTCs to ensure sensitivity and specificity.

Recent studies have shown that the expression of CTC PD-L1 can predict the prognosis of various solid tumors^42^. Papadaki et al.^43^ detected the expression of PD-L1 on CTCs in the peripheral blood of patients with breast cancer, confirming the expression of the PD-L1 protein on the CTC cell membrane and verifying the feasibility of CTC PD-L1 detection. Winograd et al.^44^ studied the effect of CTC PD-L1 expression on the prognosis of 87 patients with hepatocellular carcinoma and found that PD-L1+ CTC patients had significantly shorter OS than PD-L1− CTC patients, and CTC PD-L1 was an independent prognostic factor. Liu et al.^45^ studied the expression of PD-L1 in peripheral blood CTCs of 70 patients with GC and found that PD-L1 + CTCs were significantly associated with short OS and poor curative effect, and they could be used as a clinical prognostic marker for GC. Therefore, the expression level of PD-L1 in peripheral blood CTCs has a certain predictive value for clinical prognosis. Given the limitations of tissue detection of PD-L1 expression, PD-L1 + CTCs can be used as a potential biomarker for tumor patients with a high risk of metastasis and poor prognosis; our current research on CTC PD-L1 has achieved preliminary results, but further clinical and experimental verification is necessary.

In our study, the CTC cut-off of 2 per 7.5 mL was used as a positive control. However, there is no guide or consensus to clarify the CTC-positive cut-off value. For breast cancer, five CTCs are commonly used as a proper cut-off^46^. In patients with GC, the number of CTCs is usually less than that in patients with breast cancer. In some studies on GC, Hiraiwa et al.^24^ used two CTCs, whereas Uenosono et al.^23^ used one CTC as the cut-off. Survival analysis was also evaluated using a CTC cut-off value of 1 simultaneously, and no statistically significant differences in OS and PFS were found for GC (Supplement Fig. 5). Therefore, based on most of the research methods, our study defined CTC ≥ 2 as the positive group. In later research, we will verify with other datasets to allow it to be generally acceptable.

This study has several limitations. First, this study is a retrospective, single-center study with a relatively small sample size and data bias. Thus, a further prospective clinical cohort study is necessary to explore the value of CTCs in clinical diagnosis and treatment. Second, the above-mentioned survival results may be inaccurate because of the different enrollment and follow-up times. Thus, a long-term follow-up should be continued. Third, this study lacked dynamic monitoring of CTCs because many studies have shown that dynamic monitoring of the changing trend of CTCs, combined with the clinical message, may better evaluate the efficacy and prognosis. In addition, in our study, we selected blood samples from healthy individuals and benign gastric tumor patients as controls. However, inflammatory gastric diseases such as gastritis, peptic ulcers, etc., may also release CTC into the blood, but these sample were not available at the time of this study. Therefore, in future studies, we will collect blood samples from these patients as controls to better demonstrate the specificity of NEimFISH. Lastly, the results may be interfered by blood derived genetically abnormal cells, leading to defects in the detection of FISH probes^6^. Therefore, further confirming whether there may be undetectable tumor cells by using the current CTC method is necessary. The application of CTCs in GC still faces many challenges, such as the lack of large-scale, multicenter combined prospective randomized controlled trials. In addition, CTC detection methods are diverse, and they lack standardization. The high cost of testing is also a factor limiting its widespread use. The sensitivity and specificity still need to be further improved.

Our research confirmed that CTCs were detected among patients with a large proportion of GC. The number of CTCs tends to increase with surgical and pathological disease progression, and it can be used as a predictor for DFS. Although the number of CTCs is not an independent risk factor for OS, it may be a helpful biomarker in the choice of patients who need further comprehensive treatment or would be good for radical surgery treatment. CTCs, as an emerging liquid biopsy technology, can provide comprehensive patient information. They can be used as a potential indicator of patient prognosis after GC surgery. CTCs combine tumor markers, which may help patients who need further treatment after selection.

### Core tip

Circulating tumor cells (CTCs) as a liquid biopsy have great potential use in both clinical applications and basic cancer research. In our study, we found that the CTC number tended to increase with surgical and pathological disease progression and can also predict disease-free survival. Although it is not an independent risk factor for overall survival, it may be a helpful biomarker in the choice of patients who need further comprehensive treatment or would be good for radical surgery treatment.

### Supplementary Information

Supplementary Information.

## Data Availability

Data is provided within the manuscript or supplementary information files.
