# Supplementary material for: Circulating tumor cells are a good predictor of tumor recurrence in clinical patients with gastric cancer
Source: Sci Rep. 2024 Jun 4;14:12758. doi: 10.1038/s41598-024-63305-3 (PMC11148116; doi:10.1038/s41598-024-63305-3)
Supplement: Supplementary file 1 — Supplementary Information. [file 41598_2024_63305_MOESM1_ESM.pdf]

# Supplementary data

## Circulating tumor cells are a good predictor of tumor recurrence in clinical patients with gastric cancer

Wenxing Li[1], Xin Zhang[1], Yanqi Yang[2], Jinhe Lin[1], Kai Zhou[1], Ruifang Sun[2], Chengxue Dang[1], Dongmei Diao[1]

1. Department of Surgical Oncology, Xi'an Jiaotong University Medical College First Affiliated Hospital, 277 West Yanta Road, 710061, Xi'an, Shaanxi, China

2. Department of Pathology, School of Basic Medical Sciences, Health Science Center, Xi'an Jiaotong University, Shaanxi, 710061, China

**Supplement Table1.** The number of CTCs detected in each patient. Before radical gastrectomy(n=120), after radical gastrectomy(n=40) and on disease recurrence(n=24).

| Patient number | Reccurence (1=yes) | Death (1=yes) | Preoperative CTC number | Postoperative CTC number | CTC number when reccurence |
|---|---|---|---|---|---|
| patient 1 | 0 | 0 | 0 | 2 | |
| patient 2 | 1 | 1 | 5 | | 5 |
| patient 3 | 0 | 1 | 9 | 6 | |
| patient 4 | | | 3 | | |
| patient 5 | | | 3 | | |
| patient 6 | 0 | 1 | 5 | | |
| patient 7 | 0 | 0 | 2 | 5 | |
| patient 8 | 0 | 0 | 1 | 3 | |
| patient 9 | 0 | 0 | 4 | 3 | |
| patient 10 | 0 | 0 | 0 | 1 | |

| | | | | | |
|---|---|---|---|---|---|
| patient 11 | 0 | 0 | 2 | | |
| patient 12 | 0 | 0 | 4 | | |
| patient 13 | 0 | 1 | 3 | | |
| patient 14 | 0 | 0 | 2 | 3 | |
| patient 15 | 1 | 0 | 3 | 5 | 6 |
| patient 16 | | | 2 | 2 | |
| patient 17 | 1 | 0 | 6 | | 7 |
| patient 18 | 0 | 1 | 9 | | |
| patient 19 | 0 | 0 | 2 | | |
| patient 20 | 0 | 0 | 2 | | |
| patient 21 | 1 | 1 | 4 | 6 | 8 |
| patient 22 | 0 | 0 | 1 | | |
| patient 23 | 0 | 0 | 3 | | |
| patient 24 | 1 | 0 | 4 | 3 | 4 |
| patient 25 | | | 3 | | |
| patient 26 | 0 | 0 | 2 | | |
| patient 27 | 0 | 0 | 2 | 4 | |
| patient 28 | 0 | 0 | 1 | | |
| patient 29 | 1 | 1 | 5 | | 5 |
| patient 30 | 1 | 0 | 3 | 6 | 6 |
| patient 31 | 0 | 1 | 0 | 1 | |
| patient 32 | | | 3 | | |

| | | | | | |
|---|---|---|---|---|---|
| patient 33 | 0 | 0 | 7 | 4 | |
| patient 34 | 0 | 1 | 7 | | |
| patient 35 | | | 2 | | |
| patient 36 | 0 | 0 | 3 | 3 | |
| patient 37 | | | 3 | | |
| patient 38 | 1 | 1 | 5 | | 5 |
| patient 39 | 0 | 0 | 1 | 3 | |
| patient 40 | 0 | 0 | 2 | 2 | |
| patient 41 | 0 | 0 | 6 | | |
| patient 42 | 0 | 0 | 2 | | |
| patient 43 | 0 | 0 | 0 | 1 | |
| patient 44 | 1 | 1 | 9 | | 9 |
| patient 45 | 0 | 0 | 2 | | |
| patient 46 | 0 | 0 | 3 | | |
| patient 47 | 0 | 0 | 2 | | |
| patient 48 | 0 | 0 | 2 | 1 | |
| patient 49 | 1 | 0 | 7 | | 8 |
| patient 50 | 0 | 0 | 2 | | |
| patient 51 | 0 | 1 | 3 | | |
| patient 52 | 0 | 0 | 3 | 2 | |
| patient 53 | 0 | 0 | 1 | | |
| patient 54 | 0 | 0 | 0 | 1 | |

| | | | | | |
|---|---|---|---|---|---|
| patient 55 | 0 | 0 | 2 | | |
| patient 56 | 0 | 0 | 4 | | |
| patient 57 | 0 | 1 | 0 | | |
| patient 58 | 1 | 0 | 2 | | 4 |
| patient 59 | 0 | 0 | 2 | | |
| patient 60 | 0 | 1 | 1 | | |
| patient 61 | 0 | 0 | 0 | 0 | |
| patient 62 | 0 | 0 | 0 | | |
| patient 63 | | | 4 | | |
| patient 64 | 0 | 0 | 1 | 0 | |
| patient 65 | 1 | 0 | 5 | | 8 |
| patient 66 | 1 | 0 | 3 | 8 | 8 |
| patient 67 | 0 | 0 | 1 | | |
| patient 68 | 0 | 0 | 1 | | |
| patient 69 | 0 | 0 | 2 | | |
| patient 70 | 1 | 1 | 7 | | 7 |
| patient 71 | 0 | 0 | 2 | 2 | |
| patient 72 | 0 | 0 | 5 | | |
| patient 73 | 0 | 0 | 5 | | |
| patient 74 | 0 | 0 | 3 | 2 | |
| patient 75 | 0 | 0 | 1 | | |
| patient 76 | 0 | 0 | 2 | 1 | |

| | | | | | |
|---|---|---|---|---|---|
| patient 77 | 0 | 0 | 4 | | |
| patient 78 | 0 | 0 | 1 | | |
| patient 79 | 0 | 0 | 0 | | |
| patient 80 | 1 | 1 | 5 | 9 | 9 |
| patient 81 | 0 | 0 | 4 | | |
| patient 82 | 0 | 0 | 1 | | |
| patient 83 | 0 | 0 | 1 | 1 | |
| patient 84 | 0 | 0 | 1 | | |
| patient 85 | 0 | 0 | 3 | | |
| patient 86 | 0 | 0 | 1 | 0 | |
| patient 87 | 0 | 1 | 1 | | |
| patient 88 | 1 | 1 | 3 | | 3 |
| patient 89 | 1 | 1 | 0 | | 2 |
| patient 90 | 0 | 0 | 2 | | |
| patient 91 | 0 | 0 | 1 | 3 | |
| patient 92 | 1 | 1 | 6 | 4 | 4 |
| patient 93 | 1 | 1 | 2 | | 4 |
| patient 94 | 0 | 0 | 2 | | |
| patient 95 | 0 | 0 | 0 | | |
| patient 96 | 1 | 1 | 5 | 5 | 6 |
| patient 97 | 0 | 0 | 1 | | |
| patient 98 | 0 | 0 | 3 | | |

| | | | | | |
|---|---|---|---|---|---|
| patient 99 | 0 | 0 | 1 | | |
| patient 100 | 0 | 0 | 2 | 1 | |
| patient 101 | 1 | 1 | 5 | | 5 |
| patient 102 | 0 | 0 | 1 | | |
| patient 103 | 0 | 0 | 1 | | |
| patient 104 | 0 | 1 | 0 | | |
| patient 105 | 0 | 0 | 6 | 2 | |
| patient 106 | 0 | 0 | 0 | | |
| patient 107 | 0 | 0 | 5 | | |
| patient 108 | 0 | 0 | 0 | | |
| patient 109 | 0 | 0 | 2 | | |
| patient 110 | 1 | 0 | 0 | 0 | 2 |
| patient 111 | 0 | 0 | 2 | | |
| patient 112 | 1 | 1 | 6 | | 7 |
| patient 113 | 0 | 0 | 2 | 2 | |
| patient 114 | 0 | 0 | 1 | | |
| patient 115 | 0 | 0 | 1 | | |
| patient 116 | 0 | 0 | 0 | | |
| patient 117 | 0 | 0 | 1 | | |
| patient 118 | 1 | 0 | 0 | 1 | 3 |
| patient 119 | 0 | 0 | 0 | 1 | |
| patient 120 | 0 | 0 | 0 | | |

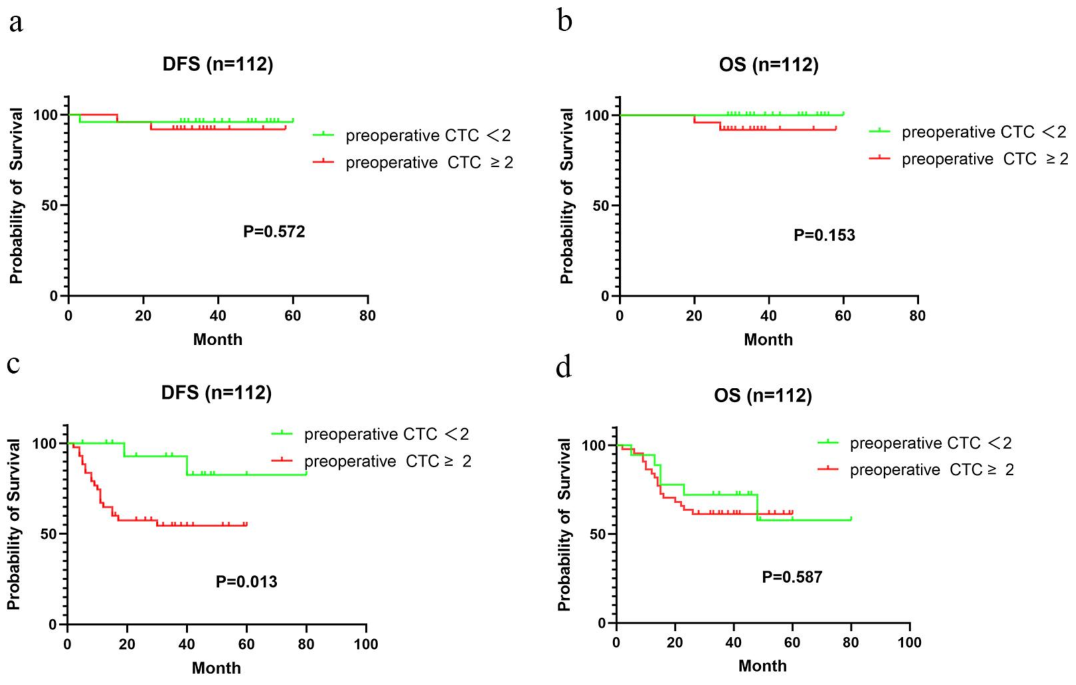


**Supplement Figure 1: Subgroup analysis of TNM staging in stages I-II and III for DFS and OS in gastric patients with preoperative CTC < 2 and CTC ≥ 2 by Kaplan-Meier survival curve. TNM stage I-II for DFS (a) and OS (b). TNM stage III for DFS (c) and OS (d).**

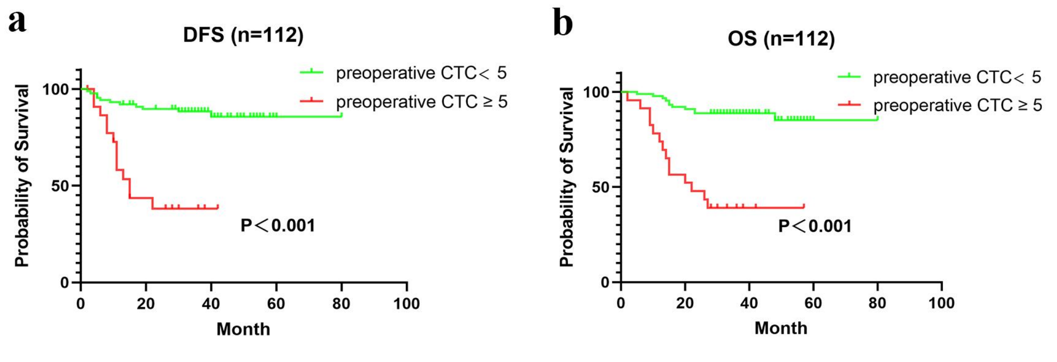


**Supplement Figure 2: Kaplan–Meier survival curves of postoperative CTC＜5 and CTC ≥ 5 groups for DFS (a) and OS (b).**

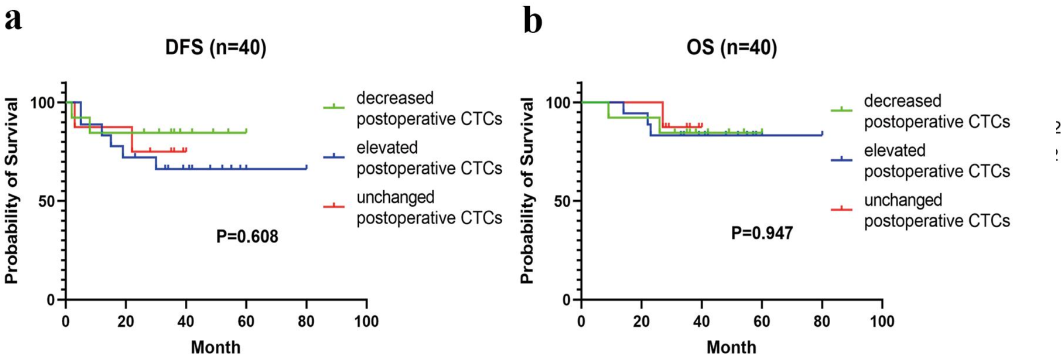


**Supplement Figure 4: Kaplan–Meier survival of dynamic changes in postoperative CTC for DFS (a) and OS (b).**

**s for**

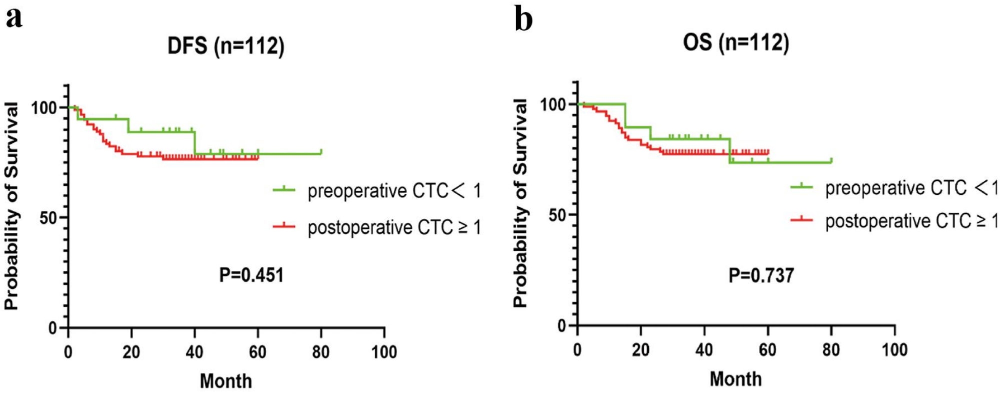


**Supplement Figure 5: Kaplan–Meier survival curves of preoperative CTC＜1 and CTC ≥ 1 groups for DFS (a) and OS (b)。**
